# Esophageal Button Battery Retrieval: Time-In May Not Be Everything

**DOI:** 10.7759/cureus.58327

**Published:** 2024-04-15

**Authors:** Sriya Bhumi, Sheena Mago, Marianna G Mavilia-Scranton, John W Birk, Houman Rezaizadeh

**Affiliations:** 1 Gastroenterology and Hepatology, New York Presbyterian, Queens, USA; 2 Gastroenterology and Hepatology, Allegheny Health Network, Pittsburgh, USA; 3 Gastroenterology-Hepatology, Connecticut GI, Farmington, USA; 4 Gastroenterology and Hepatology, University of Connecticut Health, Farmington, USA

**Keywords:** timing of endoscopy, deliberate foreign body ingestion, button battery ingestion, foreign body retrieval, esophagogastroduodenoscopy (egd), egd

## Abstract

The management of ingested foreign bodies is a challenging task because each case is unique with multiple varying factors including a patient’s age, anatomical considerations, clinical presentation, and the type and location of the foreign body ingested. Additionally, concern over complications associated with button battery ingestion typically drives management decisions. The common practice is the urgent retrieval of the foreign body within two to six hours of presentation. An unusual case is presented here that demonstrated significantly delayed endoscopic removal of an ingested button battery without complication, avoiding the many risks associated with any emergent endoscopic procedure. However, this practice is a case-by-case decision because there is a lack of literature to guide the current management.

## Introduction

The management of ingested foreign bodies is a challenging task because each case is unique with multiple varying factors including the patient’s age, anatomical considerations, clinical presentation, and the type and location of foreign body ingested. Ingested foreign bodies can cause a multitude of complications and may be retained for a prolonged period. The overall incidence of button battery ingestions is low; however, it has increased with the rise in their use [1]. The hypothesized mechanism of injury caused by ingested button batteries is multifactorial. Injury may occur from the direct pressure, electrical discharge, corrosive contents, and metal toxicity [1,2].

Emergent esophagogastroduodenoscopy (EGD) is recommended for patients who have ingested button batteries because of the higher risk of tissue damage [2,3]. The European Society of Gastrointestinal Endoscopy (ESGE) recommends endoscopic retrieval within two to six hours of presentation [4]. However, EGD within 12 to 24 hours is recommended for the ingestion of magnets or objects greater than 6 cm in length [3]. Accordingly, conservative management with close monitoring can be instituted for blunt foreign bodies that are less than 6 cm in length and 2.5 cm in diameter or those that are postpyloric [3]. Esophageal button battery impactions have been associated with esophageal fistula formation to the trachea or aorta, esophageal perforation, empyema, and vocal cord paralysis, which have been reported to occur within as little as two hours following their ingestion [2,3]. Patients who have ingested button batteries that were 20 to 25 mm large were noted to have clinically more significant adverse events compared to those who ingested 15 to 18 mm cells [2,5]. With these associated complications, esophageal button battery cases are handled emergently; however, we present an unusual case of a significantly delayed endoscopic removal without an associated complication.

This article was previously presented at a meeting as an abstract at the 2022 American College of Gastroenterology Annual Scientific Meeting and Postgraduate Course in Charlotte, NC, on October 25, 2022.

## Case presentation

A 45-year-old male with no significant past medical history presented to the hospital four days after the unwitnessed ingestion of “two magnets,” thought to have been consumed for secondary gain. Since the ingestion, he had been having difficulty eating solid foods, but he was able to tolerate liquids and handle oral secretions. He had dysphagia, but he denied any abdominal pain, neck pain, acid reflux, nausea, vomiting, black tarry stools, bright red blood per rectum, chest discomfort, drooling, wheezing, coughing, or choking. He did not have a history of tobacco, drug, or alcohol use, and he had no family or personal history of gastrointestinal (GI) cancers.

**Figure 1:**
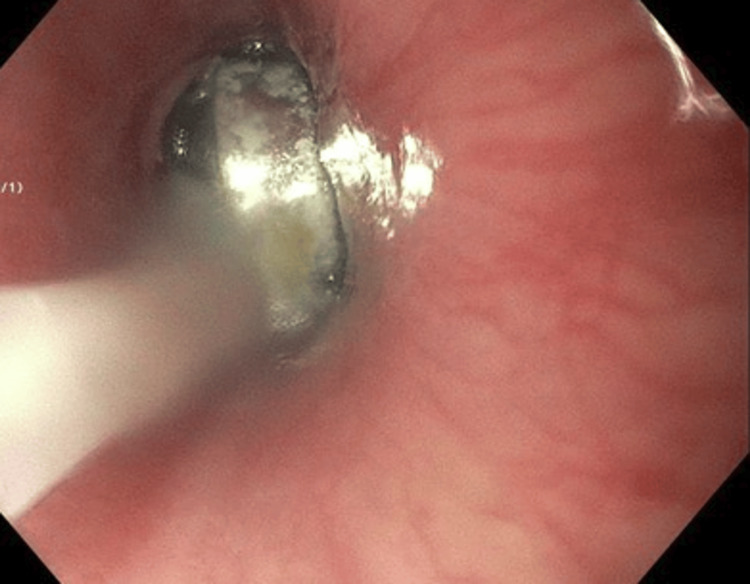
Identification of a 20 mm button battery in the upper esophagus

**Figure 2:**
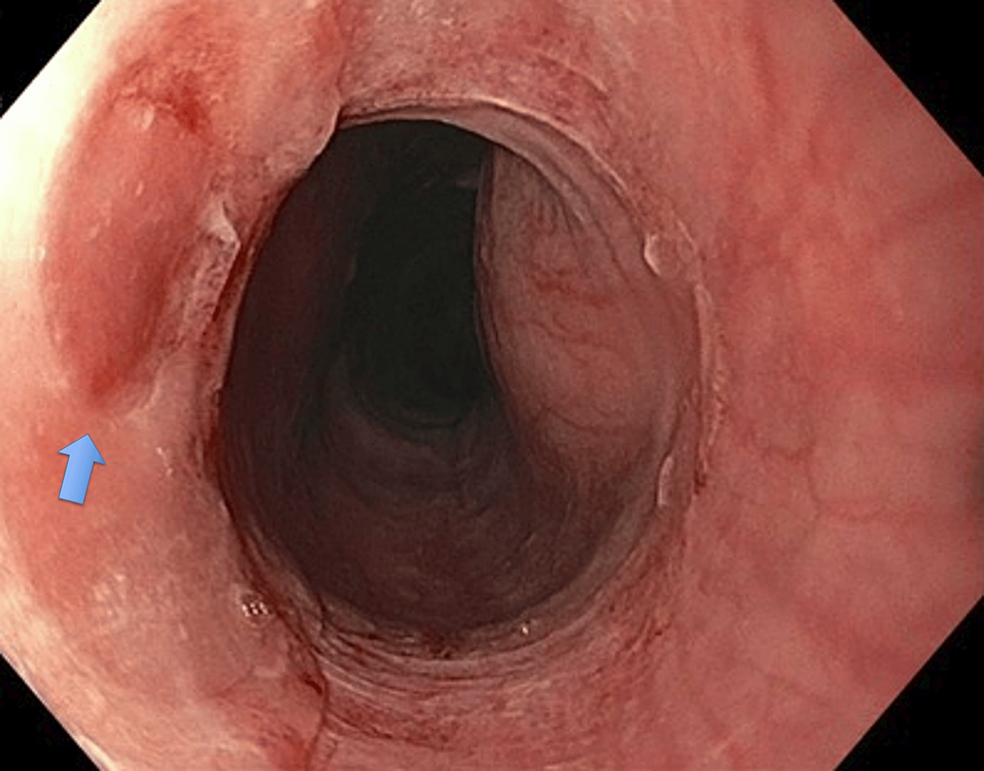
Mild localized injury of the esophageal mucosa directly underlying the object

Upon admission to the hospital, he was afebrile and hemodynamically stable and did not require any supplemental oxygenation. His abdomen was soft, non-distended, and non-tender to palpation. The foreign body could not be visualized in his oropharynx. Moreover, laboratory tests did not demonstrate evidence of leukocytosis or anemia. Radiographs of his neck, chest, and abdomen were done demonstrating one radiopaque foreign body in the proximal thoracic esophagus at the T1 level and another in the rectum. Given the patient’s refusal to undergo an endoscopic procedure, the removal of the esophageal object was delayed an additional two days. On day six, he finally agreed to have an EGD, and X-rays showed that they were still present in the esophagus. Despite the patient’s claim to have swallowed a magnet, a 20 mm button battery was identified in the upper esophagus at the level of the cricopharyngeus and was removed with a Roth net (STERIS, Mentor, USA) (Figure 1). Mild localized injury of the mucosa was noted directly underlying the object (Figure 2). The rest of the esophagus, stomach, and duodenum had no abnormalities. He was able to resume oral intake that evening without any complications.

## Discussion

The management of foreign bodies is a challenging endoscopic dilemma given the technical difficulties of endoscopic retrieval and unpredictable outcomes. Despite the increasing incidence of button battery ingestions, there is a paucity of data, particularly in adult patients, to guide management [6]. As seen in this case, the excessive duration of six days where the button battery remained within the patient’s esophagus did not correlate to the severity of injury and complications expected. Despite its larger diameter of 20 mm, the patient was noted to only have mild localized injury of the mucosa. This case suggests that esophageal dwell time alone is not an independent risk factor and may not correlate with the severity of mucosal injury.

## Conclusions

There are many risks associated with performing emergent endoscopic procedures. These include limited availability of well-trained staff during on-call hours, abbreviated time for full preoperative evaluation of the patient, and other logistic challenges. For this reason, it is important to identify situations where procedures can be scheduled and performed under controlled circumstances. Our case demonstrates that the delayed removal of an esophageal button battery did not cause serious mucosal injury and that time alone may not be an independent risk factor. However, we do caution that delayed removal of a button battery should not be considered the standard of care because of the current paucity of supportive evidence.
